# Lightning Conductors and Their Tests

**Published:** 1914-04-18

**Authors:** 


### PRACTICAL POINTS IN HOSPITAL MECHANICS.

### Lightning Conductors and their Tests.

The following is a summary of a paper recently read by Mr. Frederic H. Taylor, Assoc.M.Inst.E.E., at the Junior Institution of Engineers :—

The subject of lightning conductors is one which is, or certainly should be, of great interest to everyone, and particularly, I think, to engineers and architects. The invention of the lightning conductor is commonly attributed to Benjamin Franklin, who is said to have erected one upon his own house in Philadelphia in 1752. He is credited with having conducted the experiment of flying a kite during a thunderstorm and thus seeking to convey the lightning discharge to earth.

The Lightning Research Committee during the three years 1901-1904 were advised of over 500 cases of buildings in Great Britain damaged by lightning, or an average of 166 per annum. When buildings provided with lightning-conductor systems are occasionally struck, the explanation which appears to be justified by modern knowledge is either that the system was in bad condition or inadequate.

It is a fallacy to suppose that only very tall buildings need protection. A building may have two chimney-stacks—a tall one and a shorter one. Where the former has been fitted with a conductor the latter has been known to be struck, and the reason has been definitely ascertained.

As to the purely scientific aspect of a lightning discharge, the engineer's lot is more often that of dealing with facts than of finding scientific theories for their occurrence. The generally accepted opinion, however, is that lightning discharges are of two distinct kinds, named by Sir Oliver Lodge the "A" flash and the "B" flash respectively. The "A" flash is simple in character, and consists of a discharge between an electrically-charged cloud which is approaching the surface of the earth without any intermediate cloud intervening. The path of the "A" flash is more or less prepared by the electrostatic conditions of the case, and it is said to strike pointed conductors in preference to others.

The "B" flash is a disruptive discharge of great suddenness, and arises where another cloud intervenes between the cloud carrying the primary charge and earth, the two clouds acting like a condenser.

Lightning discharges are sometimes quite silent, and, being so, may pass unobserved.

Lightning is stated by many authorities to select the path of least resistance. But what forms the path of least resistance the lightning alone decides.

Protection against the "A" type of flash is fairly easy, and it is considered that the ordinary system of conductors, when properly constructed and maintained, is reasonably safe. Discharges of the "B" type involve more complex treatment, and in the words of the Lightning Research Committee, 1901-1905, "absolute protection can only be assured by a complete wirework enclosure; in fact, of the nature of a bird-cage." In any case, the extent to which a building shall be protected and the cost of such protection usually have to bear a certain relation to the importance and the first cost of the building itself. Unfortunately, the lightning-conductor work of most buildings is treated with an indifference as dangerous as it is unjustifiable. It is, indeed, regrettable that insurance companies have not so far, by united action, found it possible to insist upon both adequate protection and maintenance.

### Parts and Design of Conductor Systems.

Briefly put, any lightning-conductor system comprises : (1) The air terminal, consisting of what is called the "elevation rod," the top of which is provided with one, three, four, or even five points; (2) the conductor from the elevation rod to the ground, with branch conductors from other parts of the building; (3) the earth connection. The metal most commonly—in fact, almost universally—used is copper, in the form of copper tape, from, say, $1\frac{1}{2}$ in. by $\frac{1}{8}$ in. down to $\frac{1}{2}$ in. by $\frac{1}{8}$ in. Iron is electrically efficient, but is more subject to corrosion, and not so easily fitted into position. For rough guidance, however, a few general first principles may here be given.

(1) Conductors should run in as direct a line to earth as possible. Some persons prefer to keep them a certain distance from the wall. (2) Vertical rods should preferably be connected by horizontal conductors. (3) Roof metalwork, etc., should be connected to the system. (4) Chimneys should be protected, as being specially liable to be struck, due, of course, to the column of hot gases, which are good conductors, the carbon lining, and the mass of metalwork at the base. (5) Any joints in conductors should be both soldered and screwed together. (6) Conductors should be kept as much away from interior gas pipes or electric pipes, etc., as possible. (7) Similar metals should be used throughout—*i.e.*, the holdfasts should be of gunmetal, and screws or rivets, etc., of copper. (8) Several short points along a ridge are better than a few tall ones.

The earth connection of any lightning-conductor system, however simple that system may be, is of the very utmost importance. If this be inefficient, the whole system may be regarded more as a danger than as a protection. The matter of obtaining a good earth connection is mainly a question of surface contact between the conductor and moist earth. The usual method of forming an earth connection is by means of a copper plate. The thickness is not important, provided it is sufficient to withstand oxidisation. It is absolutely necessary that the earth connection should be kept fairly moist, so as to allow the resistance to remain moderate in value. It is usual to bury a quantity of broken coke or cinders with the plate, with a view to retaining the moisture. If either material be used it must be thoroughly washed so as to get rid of any sulphur present, which would destroy the copper. In order to facilitate the provision of a proper earth terminal the patent tubular earth has been designed by Mr. Killingworth Hedges, M.I.C.E.

The reasons which call for regular inspection and test are obvious : The air terminals are the most exposed part of the structure; decay of the earth-plate may arise when least suspected, and particularly disconnection just about the ground line.

Methods of testing I will divide under two heads : (*a*) the bell test, which merely shows continuity or otherwise, and (*b*) measurement of resistance test. The first method is of little or no service whatever, though it is useful as a preliminary. The measurement of resistance is mostly made by some form of Wheatstone bridge, the most usual, perhaps, being an ordinary P.O. bridge, worked with a few dry cells. The prevalence of "earth currents," due to countless telephone and similar circuits, has within my experience made this type of apparatus very troublesome, and in some cases impossible to work with.

